# A high-throughput method for detection of DNA in chloroplasts using flow cytometry

**DOI:** 10.1186/1746-4811-3-5

**Published:** 2007-03-23

**Authors:** Beth A Rowan, Delene J Oldenburg, Arnold J Bendich

**Affiliations:** 1 Department of Biology, University of Washington, Seattle, WA 98195, USA

## Abstract

**Background:**

The amount of DNA in the chloroplasts of some plant species has been shown recently to decline dramatically during leaf development. A high-throughput method of DNA detection in chloroplasts is now needed in order to facilitate the further investigation of this process using large numbers of tissue samples.

**Results:**

The DNA-binding fluorophores 4',6-diamidino-2-phenylindole (DAPI), SYBR Green I (SG), SYTO 42, and SYTO 45 were assessed for their utility in flow cytometric analysis of DNA in Arabidopsis chloroplasts. Fluorescence microscopy and real-time quantitative PCR (qPCR) were used to validate flow cytometry data. We found neither DAPI nor SYTO 45 suitable for flow cytometric analysis of chloroplast DNA (cpDNA) content, but did find changes in cpDNA content during development by flow cytometry using SG and SYTO 42. The latter dye provided more sensitive detection, and the results were similar to those from the fluorescence microscopic analysis. Differences in SYTO 42 fluorescence were found to correlate with differences in cpDNA content as determined by qPCR using three primer sets widely spaced across the chloroplast genome, suggesting that the whole genome undergoes copy number reduction during development, rather than selective reduction/degradation of subgenomic regions.

**Conclusion:**

Flow cytometric analysis of chloroplasts stained with SYTO 42 is a high-throughput method suitable for determining changes in cpDNA content during development and for sorting chloroplasts on the basis of DNA content.

## Background

Chloroplasts arose from endosymbiosis between a cyanobacterium and a eukaryotic host and contain their own genome [1]. The chloroplast genome is a remnant of the original endosymbiont, a result of gene transfer to the nucleus over evolutionary time [2] and contains fewer than 100 protein-encoding genes in land plants [3]. The number of copies of the genome per chloroplast is not constant, but changes dramatically during development. The amount of DNA per plastid has been studied by fluorescence microscopy using 4',6-diamidino-2-phenylindole (DAPI). Within chloroplasts, the DNA is associated with RNA and proteins in complexes known as nucleoids [4]. In meristematic cells, chloroplast DNA (cpDNA) replication outpaces chloroplast division, leading to an increase in the amount of DNA per chloroplast [5], possibly to allow increased capacity for protein synthesis by providing a higher dosage of rRNA genes [6]. During the cell expansion phase that follows cell division in the meristem, the amount of cpDNA is diluted by chloroplast division without compensatory DNA synthesis [7,8], accompanied by cpDNA degradation in barley [9] and rice [10]. The fate of cpDNA in mature leaves had received little attention until recently, when it was shown that cpDNA declines somewhat during development in pea and *Medicago truncatula *[11], and much more extensively in Arabidopsis [12] and maize [13]. The reduction of cpDNA occurs long before the onset of senescence in photosynthetically active leaves. Whether the loss of cpDNA is a general feature of development among all chloroplast-containing organisms remains to be assessed, in part because the assay for DNA content of individual chloroplasts is quite laborious.

The currently available methods for analyzing cpDNA content during development are time-consuming. Without a high-throughput method, it will be difficult to further investigate this developmental process and to screen large numbers of plants for mutations that affect the persistence of cpDNA. Flow cytometry is a rapid assay that has been used in a variety of applications including analysis of nuclear DNA content [14], detection of immunostaining [15], and cell viability [16]. In this study, we show that flow cytometry can be used to analyze cpDNA content during development, and to sort chloroplasts on the basis of DNA content.

## Results

### Flow cytometry using DAPI

**Figure 1:**
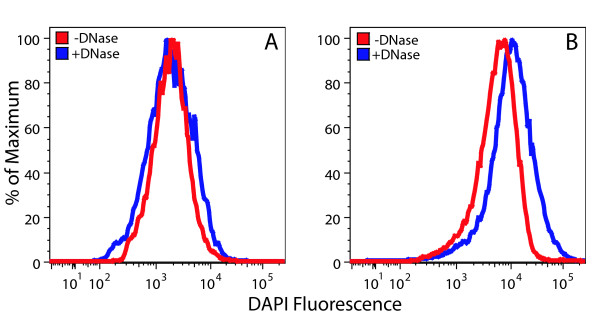
Flow cytometric analysis of DAPI-stained chloroplasts. Chloroplasts isolated from seedlings at 14 (**A**) and 20 (**B**) days after imbibition were fixed in glutaraldehyde before being treated with and without DNase and stained with DAPI. The number of chloroplasts analyzed in (**A**) was 8800 for DNase-treated and 9000 for untreated samples. The corresponding numbers in (**B**) were 23,300 and 25,300.

DAPI is a DNA-binding fluorophore commonly used for fluorescence microscopy and flow cytometry [17-20]. In this study, we have assessed its utility for flow cytometric analysis of the DNA content of Arabidopsis chloroplasts that were isolated using a high salt procedure that avoids the use of DNase during the isolation of chloroplasts [11]. After compromising the chloroplast envelope by glutaraldehyde fixation, we treated chloroplasts with and without DNase in order to detect a DNA-specific signal. We then analyzed DNase-treated and untreated chloroplasts by flow cytometry after staining with DAPI. DAPI fluorescence did not accurately reflect cpDNA content, as chloroplasts treated with DNase showed a distribution of fluorescence values that was similar to (Figure 1A) and sometimes exceeded (Figure 1B) untreated chloroplasts. Neither varying the concentration of DAPI (5 – 30 μg/mL) nor adjusting the voltage of the DAPI detection channel resulted in a fluorescence signal attributable to cpDNA (data not shown). We conclude that DAPI is not useful for flow cytometric analysis of cpDNA content.

### Flow cytometry and fluorescence microscopy of chloroplasts using SG

**Figure 2:**
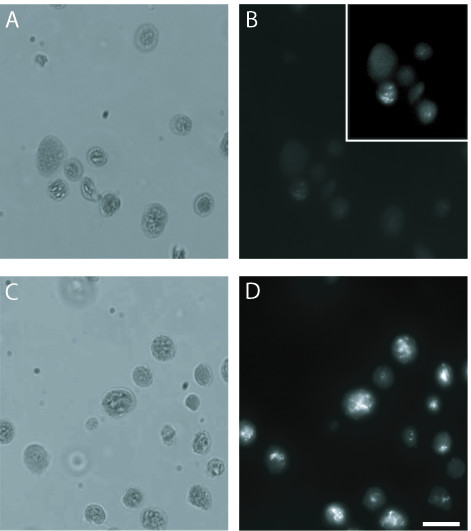
Comparison of SG and DAPI using fluorescence microscopy. Chloroplasts isolated from seedlings at 20 days after imbibition stained with DAPI (**B**) or SG (**D**). (**A**, **C**) Brightfield images of the chloroplasts shown in (**B**) and (**D**). The contrast has been enhanced (inset in (**B**)) to accentuate fluorescence from the chloroplasts in the lower left quadrant. Scale bar is 10 μm. The exposure time for both (**B**) and (**D**) was 0.5 s. DAPI-stained chloroplasts were fixed in glutaraldehyde and SG-stained chloroplasts were not fixed.

SYBR Green I (SG; excitation maximum 497 nm, emission maximum 520 nm) is a DNA-binding fluorophore commonly used in many applications, such as real-time quantitative PCR (qPCR) and staining DNA in gels [21,22]. It has been used for detection of DNA in bacteria and viruses using flow cytometry [23,24]. Chloroplasts stained with 3.5 μg/mL SG and examined by fluorescence microscopy (Figure 2D) exhibited brighter fluorescence than chloroplasts stained with DAPI (Figure 2B). Fluorescence of SG-stained chloroplasts was about 10-fold brighter at the same exposure time (0.5 s) as DAPI-stained chloroplasts from the same tissue sample.

**Figure 3:**
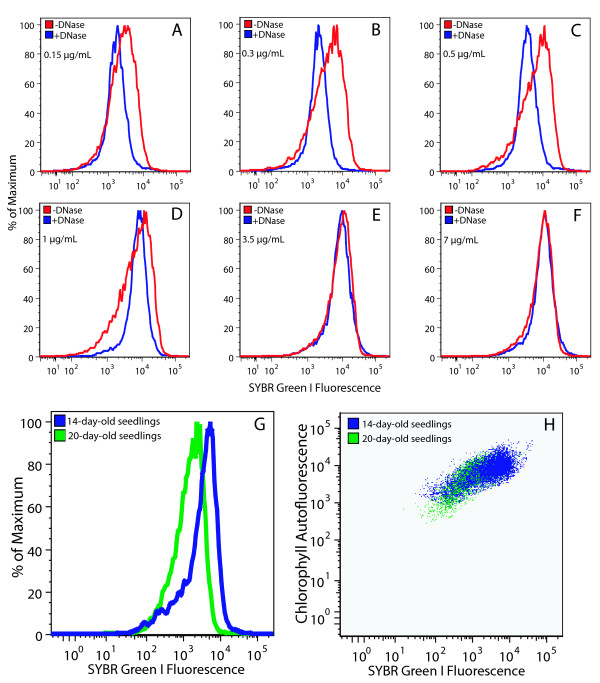
Flow cytometric analysis of SG-stained chloroplasts. (**A**-**F**) Chloroplasts isolated from 20-day-old seedlings treated with and without DNase and stained with the indicated concentrations of SG. Differences in cpDNA content (**G**) and PPoD (**H**) for chloroplasts isolated from seedlings at 14 and 20 days after imbibition. (**A**-**F**) Chloroplasts were fixed in glutaraldehyde. (**G **and **H**) Chloroplasts were not fixed. At least 6000 chloroplasts were analyzed for all samples.

Flow cytometric analysis of chloroplasts treated with or without DNase was performed over a 47-fold SG concentration range (Figure 3A–F). At lower concentrations (0.15 – 0.5 μg/mL), flow cytometric analysis revealed a fluorescence signal attributable to cpDNA, as indicated by the shift of the distribution curve of DNase-treated chloroplasts to the left (Figure 3A–C). At higher concentrations of SG (3.5 and 7 μg/mL), a DNA-specific signal was not detected (Figure 3E and 3F). Further analysis of cpDNA content was conducted at a concentration of 0.5 μg/mL SG, as this concentration showed the maximum difference between chloroplasts with and without DNA. Chloroplasts isolated at different stages of development and treated with DNase exhibit distributions that do not differ from one another, indicating a constant background fluorescence among the samples (data not shown).

Flow cytometric analysis of cpDNA can be performed on chloroplasts with or without chemical fixation. Glutaraldehyde fixation was used to render the chloroplast membrane permeable to DNase in order to provide controls for selecting the optimal dye concentration. However, glutaraldehyde fixation can also cause autofluorescence [25] not due to DNA. We compared unfixed chloroplasts to chloroplasts fixed with either glutaraldehyde or formaldehyde. We found that fluorescence of SG-stained chloroplasts increased slightly after fixation with glutaraldehyde (~12%), but decreased after fixation with formaldehyde (~17%) (data not shown). Thus, for SG, chloroplasts should either be fixed in the same manner or not be fixed.

We were able to detect differences in DNA content between chloroplasts isolated from immature and mature tissues with SG. Chloroplasts isolated from young seedlings (14 days old) had a mean fluorescence intensity of 4342, whereas those isolated from 20-day-old seedlings had a mean fluorescence intensity of 1938 (Figure 3G). In order to obtain additional information about changes that accompany chloroplast development, we analyzed SG fluorescence and chlorophyll autofluorescence simultaneously. Chloroplasts from 14-day-old seedlings had more chlorophyll, and contained a relatively high DNA content, compared with chloroplasts from 20-day-old seedlings (Figure 3G,H). In conclusion, a difference in cpDNA content between chloroplasts from different developmental stages was observed by flow cytometric analysis using SG.

### Flow cytometry and fluorescence microscopy of chloroplasts using SYTO 42

SYTO 42 is a DNA-binding fluorophore with an excitation maximum at 433 nm and an emission maximum at 460 nm. Though it is designed for fluorescence microscopy and flow cytometry, we could find no reports of its use for these applications. The Becton Dickinson LSR II that we used has a 405 nm excitation laser that excites below the maximum and an emission filter capable of detecting the emission maximum at 460 nm.

**Figure 4:**
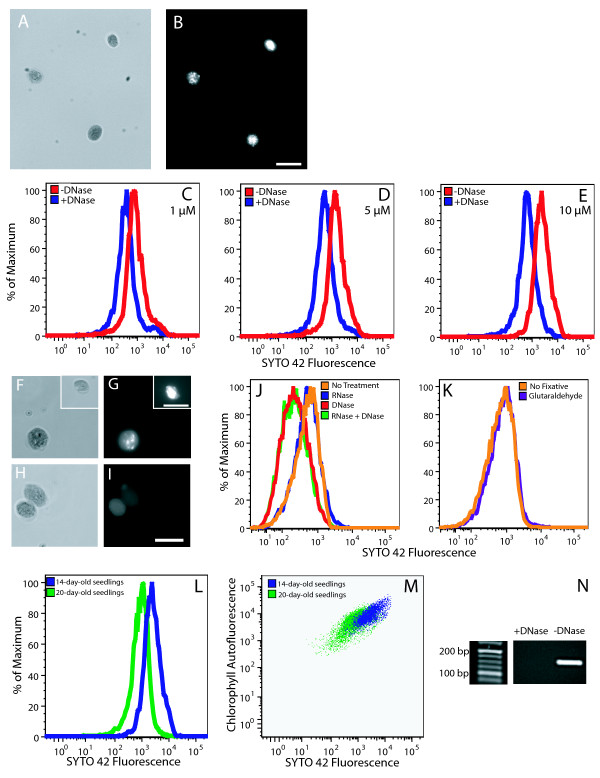
Fluorescence microscopy and flow cytometric analysis of SYTO 42-stained chloroplasts. Brightfield (**A**) and fluorescence (**B**) microscopic images of chloroplasts isolated from 14-day-old seedlings after staining with 25 μM SYTO 42. The exposure time in (**B**) was 0.1 s. (**C**-**E**) Flow cytometric analysis of chloroplasts from 14-day-old seedlings treated with and without DNase and stained with the indicated concentrations of SYTO 42. Brightfield (**F**, **H**) and fluorescence microscopy (**G**, **I**) of chloroplasts from immature leaves of 35-day-old plants after treatment with RNase (**F**, **G**) and DNase (**H**, **I**) and staining with 25 μM SYTO 42. Insets in (**F**) and (**G**) show brightfield and fluorescence images of a chloroplast from a different microscopic field of the same sample. The exposure times were 0.1 s (**G**) and 0.3 s (**I**). The number of DNase-treated chloroplasts that did not have visible nucleoids was 8 out of 8. The number of chloroplasts that did not have visible nucleoids was 1 out of 9 after RNase treatment, and 2 out of 19 for untreated controls. (**J**) Flow cytometric analysis comparing untreated chloroplasts with chloroplasts treated with RNase, DNase, or DNase and RNase from immature leaves of 35-day-old plants. (**K**) Comparison of unfixed chloroplasts to chloroplasts fixed with 0.8% glutaraldehyde. Differences in cpDNA content (**L**) and PPoD (**M**) for chloroplasts isolated from seedlings at 14 and 20 days after imbibition. (**N**) PCR amplification of a 156-bp fragment of the *psb*A gene from lysates prepared from chloroplasts that had been previously treated or not treated with DNase. Scale bars are 10 μm. (**A**, **B**, **L**, **M **(14-day profile)) Chloroplasts were not fixed. (**C**-**K**, **L**, **M **(20-day profile)) Chloroplasts were fixed in glutaraldehyde. (**C**-**E**, **J**-**M**) At least 2500 chloroplasts were analyzed for all samples.

We found that chloroplasts are permeable to SYTO 42 with or without prior fixation. Fluorescence microscopy of chloroplasts stained with 25 μM SYTO 42 showed bright staining of chloroplast nucleoids at an exposure time of 0.1 s (Figure 4B). Flow cytometric analysis of chloroplasts treated with or without DNase was performed over a concentration range from 1 μM to 20 μM SYTO 42 (Figure 4C–E and data not shown). SYTO 42-DNA fluorescence was detected at all concentrations tested, and 10 μM was selected for further use because it was the lowest concentration at which maximum separation of the distributions of chloroplasts with and without DNA was achieved.

SYTO 42 specifically reports the DNA, but not RNA, in chloroplasts. Treatment of chloroplasts with RNase did not result in a reduction of fluorescence (Figure 4G and 4J), whereas treatment with DNase did (Figure 4I,J). To assess the efficacy of our DNase treatment, chloroplasts were fixed with glutaraldehyde, washed, and treated with and without DNase, and the chloroplast lysates were used for PCR amplification. The expected band of cpDNA was detected from chloroplasts not treated with DNase (Figure 4N). The band was absent from lysates prepared from DNase-treated chloroplasts, indicating that the DNase treatment results in the complete removal of DNA from the chloroplasts. Fixation with glutaraldehyde did not increase the fluorescence of SYTO 42 (Figure 4K).

Chloroplasts isolated from 20-day-old seedlings had less DNA than chloroplasts from 14-day-old seedlings (Figure 4L,M), as expected. The mean SYTO 42 fluorescence was 3062 at 14 days after imbibition and fell to 935 by 20 days after imbibition. The difference between these two samples was 3.3-fold, compared to only 2.2-fold for the same samples analyzed using SG.

### Flow cytometry of chloroplasts using SYTO 45

SYTO 45 is a DNA-binding fluorophore that is similar to SYTO 42. We could find no reports of its application to either fluorescence microscopy or flow cytometry. SYTO 42 has a 370-fold fluorescence enhancement upon binding DNA, whereas that for SYTO 45 is 4660-fold. SYTO 45 has an excitation maximum at 455 nm and emission maximum at 484 nm. This dye was included in this study because of the high fluorescence enhancement upon binding DNA, even though the 405 nm excitation laser and 420 – 460 nm emission filter set on the Becton Dickinson LSR II flow cytometer that we used only encompass the lower ends of the excitation and emission spectra of SYTO 45.

**Figure 5:**
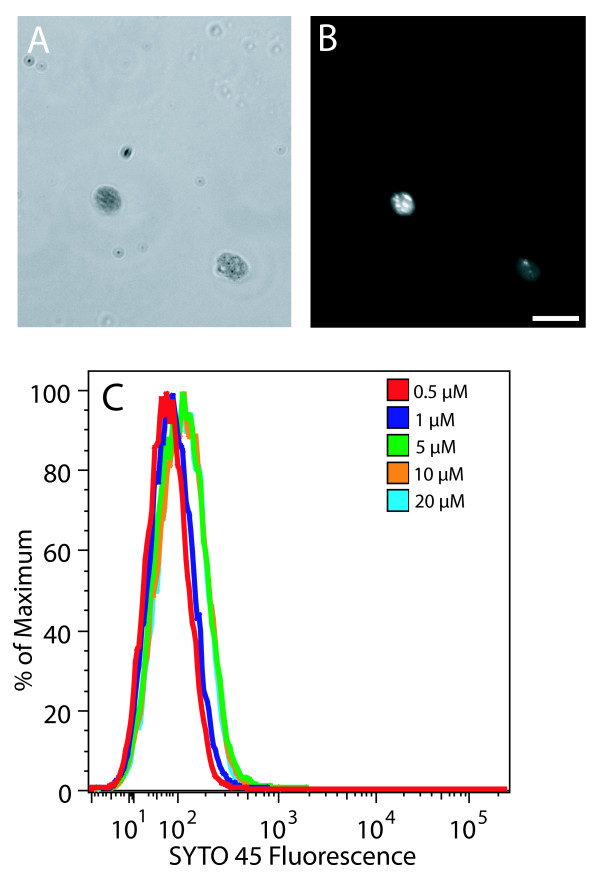
Fluorescence microscopy and flow cytometric analysis of SYTO 45-stained chloroplasts. Brightfield (**A**) and fluorescence (**B**) microscopic images of chloroplasts isolated from 14-day-old seedlings after staining with 20 μM SYTO 45. The exposure time for (**B**) was 0.1 s. (**C**) Flow cytometric analysis of the same chloroplasts stained with the indicated concentrations of SYTO 45. Scale bar is 10 μm. Chloroplasts in all panels were not fixed.

Chloroplasts stained with SYTO 45 at 20 μM exhibited bright staining of nucleoids at a 0.1 s exposure (Figure 5B) that appeared similar to that with SYTO 42 at 25 μM. We found that chloroplasts with or without prior fixation were permeable to SYTO 45. Chloroplasts stained with SYTO 45 at a range of concentrations from 1 μM to 20 μM and analyzed using flow cytometry showed only slight differences (Figure 5C). We conclude that the high fluorescence enhancement of this dye does not compensate for the sub-maximal excitation and emission capabilities of our instrument.

### Flow cytometry using SYTO 42 and a high-throughput assay of changes in cpDNA content during development

The fluorescence of DAPI-DNA was measured using the microscopic method we used previously [12] for chloroplasts isolated (without the use of DNase) from tissues at four different developmental stages. The same samples were also analyzed by flow cytometry using SYTO 42, since this fluorophore demonstrated the best detection capability. Twelve day-old Arabidopsis seedlings had cotyledons and four rosette leaves. The cotyledons were about 3 mm in length and fully expanded. The first and second rosette leaves were 3–5 mm in length and in the expansion phase of development. The third and fourth leaves were visible and just beginning to expand. Chloroplasts were isolated from the cotyledons and the first pair of rosette leaves. Twenty-three-day-old Arabidopsis plants had initiated flowering. Chloroplasts were isolated from the first and fifth rosette leaves (both fully expanded), which were approximately 10 mm and 30 mm in length, respectively.

**Figure 6:**
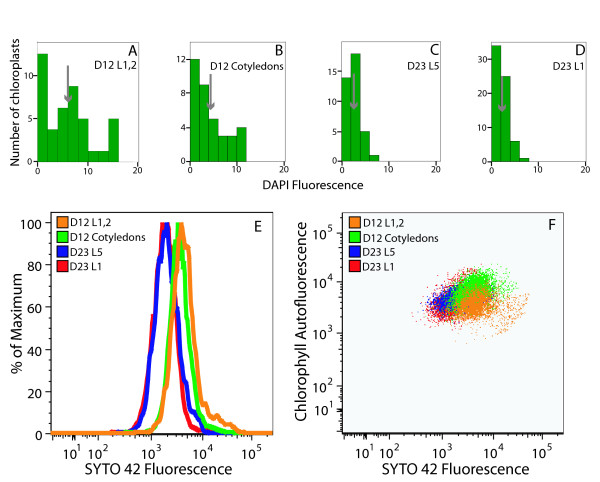
Analysis of changes in cpDNA content during development using fluorescence microscopy and flow cytometry. (**A**-**D**) The frequency of relative fluorescence values of DAPI-stained, glutaraldehyde-fixed chloroplasts isolated from four different tissues at two stages of growth measured by fluorescence microscopy. Flow cytometric analysis (**E**) and PPoD (**F**) of the same four samples shown in (**A**-**D**) using SYTO 42 (chloroplasts were not fixed). D12 L1,2: first two leaves of 12-day-old seedlings. D12 Cotyledons: cotyledons of 12-day-old seedlings. D23 L5: fifth leaf of 23-day-old plant. D23 L1: first leaf of 23-day-old plant. The means (arrows) ± standard error are 6.14 ± 0.76 (**A**), 4.39 ± 0.81 (**B**), 2.62 ± 0.2 (**C**), 2.14 ± 0.18 (**D**). The numbers of chloroplasts with no detectable DNA (and the number of chloroplasts assayed) by DAPI-staining are 0(37), 0(36), 0(62), 4(66) for the samples shown in (**A**-**D**), respectively. The means of the SYTO 42 fluorescence profiles shown in (**E**) are 5835 (D12 L1,2), 4128 (D12 Cotyledons), 2344 (D23 L5), and 2042 (D23 L1). At least 3000 chloroplasts were analyzed. Mean SYTO 42 fluorescence values were 7607, 6007, 3115, 2431 and 8471, 5440, 3523, 2549 for the same tissues obtained from the ecotypes Nossen and Estland, respectively (profiles not shown).

Analysis by fluorescence microscopy shows a developmental decline in cpDNA content (Figure 6A–D), as reported previously [12]. The mean DAPI Rfl (Relative fluorescence; see Materials and Methods) was 6.14 for chloroplasts isolated from the immature first and second rosette leaves, and fell to 2.14 in the 23-day-old first rosette leaf, but the tedious nature of the analysis restricted the number of chloroplasts analyzed (35 – 66 per sample). Flow cytometric analysis of the same chloroplast samples showed the same trend (Figure 6E), but thousands of chloroplasts were scored. The mean SYTO 42 fluorescence was 5835 for the immature first and second leaves and declined to 2042 in the 23-day-old first rosette leaf.

By simultaneously analyzing chlorophyll and DNA content using flow cytometry, it is possible to generate a Plastid Profile of Development (PPoD; Figures 3H, 4M and 6F). For the four tissues described above, the PPoD shows how chlorophyll and cpDNA content vary during leaf development. Chloroplasts from immature rosette leaves have a lower chlorophyll, but similar DNA content to chloroplasts from cotyledons of 12-day-old plants. In the leaves of 23-day-old plants, both chlorophyll and DNA content are reduced. We conclude that flow cytometry can be used to rapidly analyze DNA and chlorophyll contents of chloroplasts.

### Determination of DNA content by qPCR after fluorescence-activated cell sorting (FACS) of SYTO 42-stained chloroplasts

**Figure 7:**
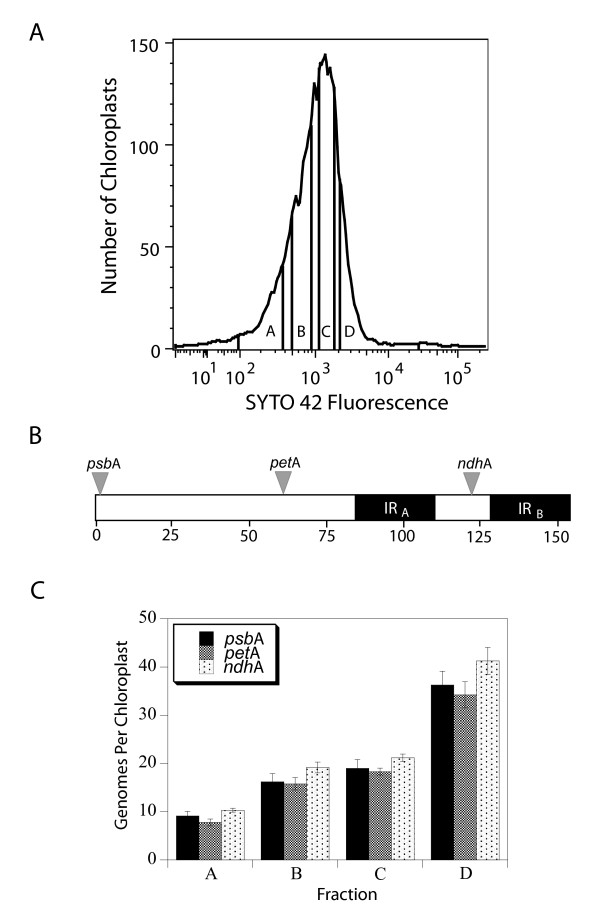
Analysis of cpDNA content by qPCR of chloroplasts obtained from fractions spanning the distribution of SYTO 42 fluorescence values. (**A**) SYTO 42 fluorescence profile of chloroplasts from mature leaves of 43-day-old plants showing the four fractions collected by FACS. Chloroplasts were not fixed. (**B**) Schematic diagram of the chloroplast genome showing the location of the primer sets used for qPCR analysis. Numbers indicate distance (in kb) on the genome map [30] (**C**) qPCR analysis of cpDNA content for the four fractions described in (**A**). Values shown are means ± standard error for 5 or 6 replicates.

Chloroplasts isolated (without the use of DNase) from mature leaves of 43-day-old plants were stained with SYTO 42 and sorted into 4 non-overlapping fractions spanning the distribution of SYTO 42 fluorescence (Figure 7A). We then used qPCR to determine the DNA content per chloroplast for each of the fluorescence intensity fractions. Figure 7C shows that, as expected, SYTO 42 fluorescence does reflect cpDNA content for each of three widely-spaced regions of the chloroplast genome (Figure 7B). Thus, it is likely that the entire genome undergoes copy number reduction during chloroplast maturation, rather than selective reduction/degradation of subgenomic regions. We conclude that the broad profile of SYTO 42 fluorescence revealed by flow cytometry does accurately reflect a broad distribution of DNA content per chloroplast and that FACS can be used to sort chloroplasts on the basis of DNA content.

## Discussion

Though the use of DAPI for the flow cytometric assessment of DNA content is well established for isolated nuclei [14], its application for the much lower DNA content of chloroplasts was not previously reported. The fluorescence of DAPI bound to DNA was much weaker and required longer exposure times for microscopic image analysis compared to the other dyes included in this study. The DAPI-DNA signal is evidently too weak to be detected in the brief time period during which DAPI-stained chloroplasts are excited by the laser in our flow cytometer.

Both SG and SYTO 42 are useful fluorophores for analysis of cpDNA content by flow cytometry. Though many cytometers are equipped to analyze SG, we recommend using SYTO 42 because it provides greater sensitivity of cpDNA detection and its fluorescence is not affected by fixation with glutaraldehyde. SYTO 42 fluorescence values correlate with differences in cpDNA content as determined by qPCR. The SYTO 45 fluorophore should provide even more sensitivity for detection of cpDNA, but the limited number of excitation wavelengths currently available for laser-excitation flow cytometers prevents this potential from being realized.

Cho et al. [26] used flow cytometry to assess DNA content of mitochondria and chloroplasts using propidium iodide (PI). The emission spectrum of PI is similar to that of chlorophyll, making it difficult to analyze cpDNA content during chloroplast development. The PPoD described in the present study can be used as a "signature" of development from proplastid to chloroplast and may be the parameter of choice when comparing plant species with respect to their relative degree of retention of cpDNA during development. The PPoD may also be useful in identifying tissues and plant growth conditions for attempting to introduce exogenous genes in chloroplast transformation experiments [27,28].

## Conclusion

We have demonstrated that flow cytometry is a rapid method to analyze DNA content of chloroplasts, and that FACS can be used to sort chloroplasts on the basis of DNA content. It takes several hours to measure DNA content of a small sample of chloroplasts (~100) by fluorescence microscopy, whereas thousands of chloroplasts can be analyzed in seconds using flow cytometry. This new technique will facilitate the analysis of the genetic and environmental aspects of the loss of cpDNA during development.

## Materials and methods

### Growth conditions

Seeds of *Arabidopsis thaliana *(Columbia) were sown on rockwool and grown at 20°C on 16 h light/8 h dark cycles. Whole plants were harvested 14 and 20 days after imbibition. Seeds of *A. thaliana *(Columbia, Nossen and Estland) were also sown in soil and grown in a greenhouse for the harvest of cotyledons and leaves. The cotyledons and first two rosette leaves were harvested 12 days after imbibition. The first and fifth rosette leaves were harvested at 23 days after imbibition. Immature leaves from 35-day-old and mature leaves from 43-day-old plants were also harvested from the Columbia ecotype.

### Chloroplast isolation

Plant tissue was soaked in 0.5% sarkosyl solution for 3–5 min, rinsed thoroughly, and ground using a mortar and pestle in 1.25 M NaCl, 40 mM HEPES, pH 7.6, 5 mM EDTA, pH 8, 0.1% bovine serum albumin, 0.1% β-mercaptoethanol following the high salt protocol [11]. This high salt method prevents contaminating DNA from adhering to the chloroplast envelope during the isolation process. The homogenate was filtered through Miracloth and centrifuged in a microcentrifuge at 12,000 × *g *for 20 s. With large volumes, the homogenate was centrifuged at 3,000 × *g *for 5 min. The pellet was resuspended and washed twice in sorbitol dilution buffer (SDB; 0.33 M sorbitol, 20 mM HEPES, 2 mM EDTA, 1 mM MgCl_2_, 0.1% bovine serum albumin [11]). The chloroplasts were loaded onto step gradients (30%:70% Percoll in SDB) and centrifuged for 10 min at 4°C in a microcentrifuge. For large volumes, the step gradients were centrifuged for 30 min at 4°C at 1,500 × *g*. The chloroplasts were taken from the 30%:70% Percoll interface and washed twice in SDB.

### Fluorescence microscopy using DAPI, SG, SYTO 42, and SYTO 45

Chloroplasts were stained with DAPI as in [12]. SYBR Green I (Molecular Probes Inc, Carlsbad, CA) was supplied by the manufacturer at a stock solution of 10,000× in DMSO. The concentration of the stock was measured [29] as approximately 10 mg/mL. Thus, the unit × represents 1 μg/mL. Concentrations of SG used in this study are reported in μg/mL. SYTO 42 and 45 (Molecular Probes, Inc, Carlsbad, CA) were supplied by the manufacturer as 5 mM solutions. Chloroplasts were adjusted to 1% β-mercaptoethanol and 3.5 μg/mL (3.5×) SG or 25 μM SYTO 42 or 20 μM SYTO 45. Samples containing 0.8% glutaraldehyde were included for comparison to unfixed samples. For SG, a sample that had been previously fixed in 3.7% formaldehyde for 1 h was also included. Images of chloroplasts were obtained according to [12]. DAPI-DNA fluorescence was measured for images taken from microscopic fields using a 345 – 375 nm excitation filter and a 435 – 485 nm emission filter. SG-DNA fluorescence was detected using a 460 – 480 nm excitation filter and a 515 nm long pass emission filter. Fluorescence of DNA bound to SYTO 42 and SYTO 45 was measured using a 426 – 446 nm excitation filter and a 465 – 495 nm emission filter. Fluorescence was analyzed on a scale of 0 – 1,023 grayscale units. The value of relative fluorescence (Rfl) for DAPI-DNA was calculated by multiplying the fluorescence intensity by the area of fluorescence and dividing by 1,000 [12].

### Treatment of chloroplasts with DNase and RNase

Chloroplasts were fixed in 0.8% glutaraldehyde for 1–2 h in order to compromise the chloroplast membranes and washed four times with SDB. Chloroplasts were then left untreated or treated with DNase (1 mg/mL DNase and 10 mM MgCl_2_), RNase (0.2 μg/mL) or DNase and RNase. Samples were incubated on ice for ~24 h before washing with SDB, staining and analysis.

### Flow cytometry of chloroplasts

Chloroplasts in SDB were stained with 5 – 30 μg/mL DAPI, 0.15 – 7 μg/mL (0.15 – 7x) SG, 1 – 20 μM SYTO 42, or 1 – 20 μM SYTO 45 for 15–30 min prior to analysis using a Becton Dickinson LSR II flow cytometer. Data were acquired using FACSDiva software and analyzed using FlowJo v. 6.3 (Treestar, Ashland, OR). DAPI was excited using a 355 nm laser and detected using a 420 – 460 nm filter set. SG was excited using a 488 nm laser and detected using a 515 – 545 nm filter set. SYTO 42 and 45 were excited using a 405 nm laser and detected using a 420 – 460 nm filter set. Chlorophyll was excited using the 488 nm laser and detected using a 663 – 677 nm filter set. Chloroplast samples treated with and without DNase were compared in order to distinguish DNA-containing chloroplasts from debris. The voltage settings were held constant when collecting the data presented as profiles within a given figure panel. Thus the fluorescence distributions within a given panel can be compared meaningfully. Chloroplasts to be compared within a single panel were fixed or not fixed with 0.8% glutaraldehyde, although the SYTO 42-DNA fluorescence profiles are indistinguishable with or without fixation (Figure 4K).

### Fluorescence-activated cell sorting (FACS) of chloroplasts

Chloroplasts were stained with 10 μM SYTO 42 and sorted using a Becton-Dickinson FACSAria cell sorter. SYTO 42 was excited using a 405 nm laser and detected with a 420–460 nm emission filter set. Four non-overlapping gates across the distribution of SYTO 42 fluorescence were used to designate the fractions to be sorted. The sorted fractions were collected into SDB to provide them with the proper osmoticum and stored on ice for further processing and analysis immediately following collection.

### Lysis of chloroplasts for PCR and real-time quantitative PCR (qPCR)

Chloroplasts were lysed in a solution containing 1% SDS, 2.5 mM EDTA, and 200 μg/mL proteinase K and incubated for at least one hour at 37°C. Proteinase K was inactivated by the addition of 0.1 mM PMSF, and 20 mM potassium acetate was added to precipitate the detergent. The lysates were centrifuged at 12,000 × g for 10 min at 2 – 8°C to remove the precipitated detergent. For the fractions collected by FACS, chloroplasts were first counted using an eosinophil counting slide (Spiers-Levy, Blue Bell, PA) before lysis was performed with a known concentration of chloroplasts. Conventional PCR amplification of a 156-bp fragment of the chloroplast *psb*A gene was performed using the forward primer 5'AGAGACGCGAAAGCGAAAG3' and reverse primer 5' CTGGAGGAGCAGCAATGAA 3'. Amplification of 1 μL template DNA was performed in a 25 μL reaction mixture containing 0.2 μM primers, 0.2 mM dNTPs, 1.5 mM MgCl_2_, and 1 unit of Taq polymerase. Following an initial denaturation at 94°C for 2 min, 32 cycles of 20 s denaturation at 94°C, 30 s annealing at 57°C, and 45 s extension at 72°C were performed using a PTC-100 programmable thermal controller from MJ Research Systems (now a division of Bio-Rad Laboratories, Hercules, CA).

For real-time qPCR, amounts of cpDNA ranging from 5 fg/μL to 50 pg/μL were used to generate a standard curve for determining the concentration of cpDNA present in the lysates of chloroplast fractions. Standards were diluted in the same solution as used for the lysates to provide identical reaction conditions for standards and unknowns. Three analyses were performed using primer sets amplifying different regions of the chloroplast genome. The forward primer 5' TTGCGGTCAATAAGGTAGGG 3' and reverse primer 5' TAGAGAATTTGTGCGCTTGG 3' were used to amplify a 189-bp fragment including part of the *psb*A gene and an intergenic region. The forward primer 5' CACCCGAGATGAAAGAAAAG 3' and reverse primer 5' AGTAGCAGGGTCTGGAGCAA 3' were used to amplify a 143-bp fragment of the *pet*A gene. The forward primer 5' TGAGATCCGCTAAAACAAGG 3' and the reverse primer 5' CTAGCCGATGGGACAAAA 3' were used to amplify a 157-bp fragment of the *ndh*A gene. Amplification of 1 μL template DNA was performed in a 25 μL reaction mixture containing 0.12 μM primers, 0.2 mM dNTPs, 4.5 mM MgCl_2_, 0.25 μg/mL SYBR Green, and 1 unit of Taq polymerase. Three replicates of each standard and five to six replicates of each sample were included in each of the three analyses. Following an initial denaturation at 94°C for 2 min, 45 cycles of 15 s denaturation at 94°C, 15 s annealing at 55°C, and 20 s extension at 72°C were performed and amplification of the reactions monitored using the Chromo 4 real-time detection system (Bio-Rad Laboratories, Hercules, CA). A melting curve from 65°C to 95°C was used to confirm the presence of single products. Data were analyzed using the Opticon Monitor 3 software (Bio-Rad Laboratories, Hercules, CA), and the amount of DNA in each of the unknown samples was determined in fg/μL. One fg represents approximately 6.33 copies of the chloroplast genome. The number of copies of the chloroplast genome per μL was calculated from the number of fg/μL divided by the number of chloroplasts per μL to obtain the number of copies of the chloroplast genome per chloroplast.

## Competing interests

The author(s) declare that they have no competing interests.

## Authors' contributions

BR conducted the chloroplast isolation and obtained the data. AB and DO participated in the experimental design and data analysis. BR and AB wrote the manuscript. All authors have read and approved the final manuscript.
